# A *Lactobacillus rhamnosus* Strain Induces a Heme Oxygenase Dependent Increase in Foxp3+ Regulatory T Cells

**DOI:** 10.1371/journal.pone.0047556

**Published:** 2012-10-15

**Authors:** Khalil Karimi, Nalaayini Kandiah, Jessie Chau, John Bienenstock, Paul Forsythe

**Affiliations:** 1 The Brain Body institute, McMaster University, Hamilton, Ontario, Canada; 2 Firestone Institute for Respiratory Health, McMaster University, Hamilton, Ontario, Canada; 3 Department of Medicine, McMaster University, Hamilton, Ontario, Canada; 4 Department of Pathology and Molecular Medicine, McMaster University, Hamilton, Ontario, Canada; McGill University Health Center, Canada

## Abstract

We investigated the consequences of feeding with a *Lactobacillus* species on the immune environment in GALT, and the role of dendritic cells and heme oxygenase-1 in mediating these responses. Feeding with a specific strain of *Lactobacillus rhamnosus* induced a significant increase in CD4+CD25+Foxp3+ functional regulatory T cells in GALT. This increase was greatest in the mesenteric lymph nodes and associated with a marked decrease in TNF and IFNγ production. Dendritic cell regulatory function and HO-1 expression was also increased. The increase in Foxp3+ T cells could be prevented by treatment with a heme oxygenase inhibitor. However, neither inhibition of heme oxygenase nor blockade of IL-10 and TGFβ prevented the inhibition of inflammatory cytokine production. In conclusion *Lactobacillus* feeding induced a tolerogenic environment in GALT. HO-1 was critical to the enhancement of Foxp3+ regulatory T cells while additional, as yet unknown, pathways were involved in the down-regulation of inflammatory cytokine production by T cells.

## Introduction

The gut microbiota impacts various aspects of host physiology including modulation of the immune system [1]–[5]. Overall balance in composition of the microbiota, together with the influence of pivotal species that induce specific responses are important determinants of immunity in the intestine and beyond. Non-pathogenic bacteria that promote beneficial health effects when ingested have been termed probiotics [6]. These “beneficial microbes” are most frequently Lactobacillus or Bifidobacterium species, however a number of lactic acid bacteria and non-pathogenic E.coli have also been identified as probiotics [7]. While initial attention was focused on the role of probiotics in gastrointestinal development, immune adaptation and attenuation of GI inflammatory diseases there is increasing interest in the ability of orally delivered probiotics to regulate immune responses outside the GI tract.

There is evidence linking the immunomodulatory function of certain commensal bacteria, and components thereof, to induction of regulatory T cells (Treg) and their associated cytokines. In vitro, selective bacteria have been shown to induce IL-10 producing Treg [8]. While, in vivo, Mazmanian et al. demonstrated that oral ingestion of polysaccharide A (PSA) derived from *Bacteroides fragilis* protects animals from experimental colitis through induction of IL-10-producing CD4+ T cells [9], [10] and dendritic cells co-cultured with PSA and then incubated with naïve T cells promoted the generation of an IL-10 producing Treg population. *Bifidobacterium infantis* induces expression of Foxp3, a key transcription factor in programming Treg, in T cells that protect mice against *Salmonella typhimurium* infection [11]. We have previously demonstrated that attenuation of the allergic airway response following oral treatment with *Lactobacillus rhamnosus* JB-1 in mice corresponded with a significant increase in the proportion of functional CD4^+^CD25^+^Foxp3^+^ Treg in the spleen and mediastinal lymph nodes [12]. Furthermore, adoptive transfer of these Treg mimicked the protection from airway inflammation observed following feeding of the bacteria. In another study of three candidate probiotic strains, the ability to promote Foxp3 expression in T cells, in mesenteric lymph nodes and spleen, was associated with protection against airway inflammation in OVA-sensitized animals and inhibition of IgE induction to orally administered OVA [13]. However, while Treg may play an important role in the anti-inflammatory response to certain commensal bacteria much remains unknown about the mechanisms underlying the induction of these cells.

Dendritic cells (DC) are among the first immune cells to encounter bacteria in the intestine and can directly present antigens from commensals to the mesenteric lymph node (MLN) [14], [15]. DC interact with T and B cells to maintain non-inflammatory immune responses through mechanisms that include direct suppression and deletion of T cells and the induction of a range of regulatory T cell subtypes [16], [17]. Thus while Treg may be major effectors of immune regulation mediated by some probiotics, the phenotypic changes in DC following interaction with bacteria are likely to be critical in orchestrating these immune responses. A range of mediators and enzymes expressed by DC, have been linked to the capacity of these cells to induce a tolerogenic or regulatory immune response including IL-10, TGFβ, indoleamine 2,3 dioxygenase (IDO) and COX-2 [18]–[20]. Recently there has been increasing interest in the immunomodulatory actions of heme oxygenase-1 (HO-1), an enzyme that catalyzes the rate-limiting step in the degradation of heme to biliverdin [21]. Antigen presenting cells (APC) expressing HO-1 promote the formation and function of Foxp3+ Treg [22] and the enzyme also plays a critical role in active immune suppression by DC [23]. Here we investigate immune responses in the GALT following oral treatment with a strain of lactobacillus, *L. rhamnousus* JB-1, previously demonstrated to have immunomodulatory actions in a number of model systems [12], [24], [25]. Further, we address the potential role for DC and HO-1 in mediating the changes induced by this organism.

## Results

### Lactobacillus Rhamnosus JB-1, but not L.salivarius, Increases Foxp3+ T cell Population in Peyer’s Patches and Mesenteric Lymph Nodes

**Figure 1:**
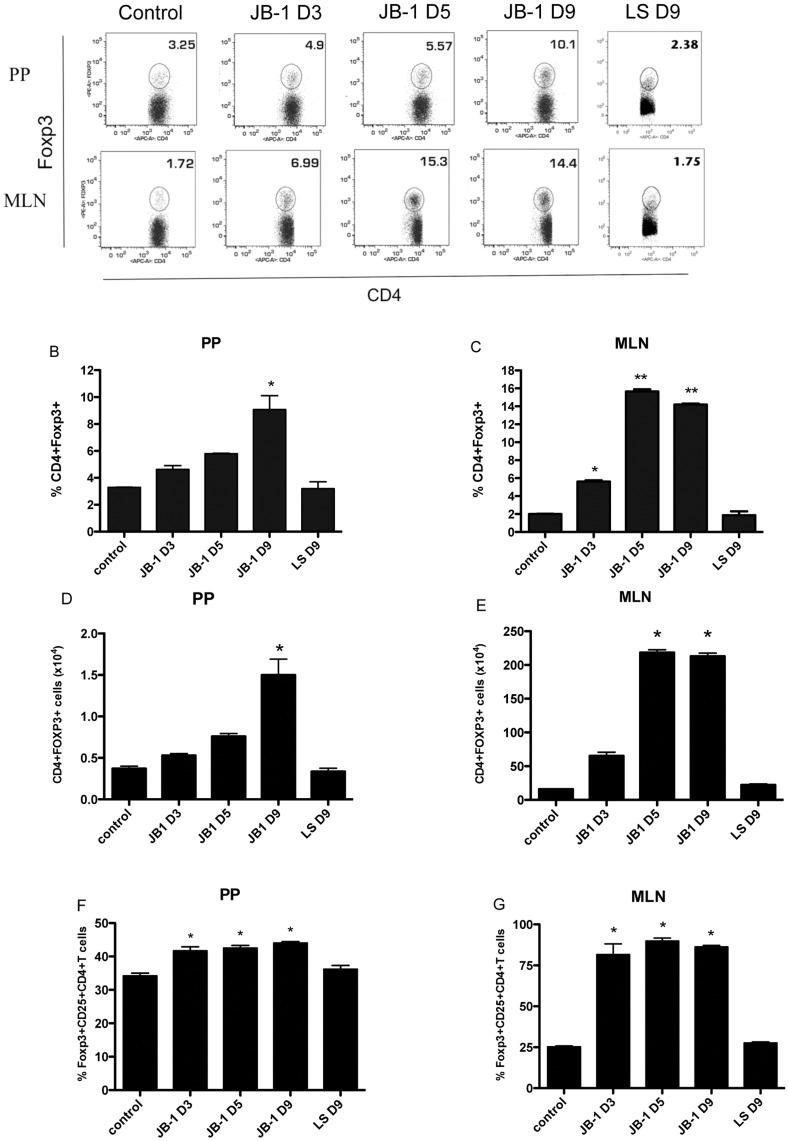
Increase in Foxp3^+^ T regulatory cells in Peyer’s patches and mesenteric lymph nodes following JB-1 feeding. Balb/c mice were given *L. Rhamnosus* JB-1, (LR) *L.salivarius* (LS) or vehicle by gavage for 3, 5 or 9 days. Single cell suspensions of Peyer’s patches (PP) or mesenteric lymph nodes (MLN) stained for CD3, CD4 and Foxp3 and analyzed by flow cytometry. Representative dot plots (A) and mean ± SEM values of Foxp3+ cells as a percentage of total (B&C) and absolute number (D&E) among the CD3+CD4+ cells and absolute number among the CD3+ CD4+ CD25+ (F&G) on days 3, 5, and 9 in lactobacillus and vehicle-fed mice are depicted. n = 5 *p<0.05.

Feeding with JB-1 lead to a significant increase in the percentage of CD4+ T cells expressing of Foxp3 in both the Peyer’s Patches (PP) and mesenteric lymph nodes (MLN). This increase was greatest in the MLN, where following 3 days of treatment the percentage of Foxp3+ CD4+ T cells increased from 1.9±0.05% to 5.6±0.1% and further increased to 15.6±0.25% and 14.2±0.1% at 5 and 9 days treatment respectively (Figure 1A,B&C) this reflected an increase in total number of Foxp3+ CD4+ cells from 15.9±0.2×10^4^ to 213±4.×10^4^ following 9 days treatment. While the Foxp3+ cell population also increased in the Peyer’s Patches this reached a statistically significant level only after 9 days of treatment (3.3±0.05% vs 9.0±1.0%, n = 5 p<0.05) with total Foxp3+ CD4+ cell numbers increasing from 0.37±0.003×10^4^ to 1.5±0.09×10^4^).

When focusing on the expression of Foxp3 in the CD4+CD25+ population there was a small, statistically significant, increase in PP but again changes were most marked in the MLN (Figure 1D&E). The increase of CD4+CD25+Foxp3+ cells at both sites was observed following 3 days treatment (34.2±0.8% vs 41.7±1.2% and 25.3±0.5% vs 81.6±6.5% in PP and MLN respectively), these enhanced levels were maintained but did not increase further with up to 9 days of treatment (figure 1 D&E). We did not observe significant changes in Foxp3 expression within the CD4+CD25– population in either the PP or MLN (data not shown). Another strain of Lactobacillus, *L.salivarius*, that we had previously demonstrated to lack some of the immunomodulatory effects of JB-1 [12], [24] did not induce increases in Foxp3 expression following up to 9 days treatment (figure 1), again suggesting that the observed effects on Foxp3 expression are dependent on strain of bacteria used.

### Lactobacillus Rhamnosus JB-1 Enhances the Suppressive Function of CD4+CD25+ Cells and Decreases Inflammatory Cytokine Production by T cells in the Mesenteric Lymph Nodes

**Figure 2:**
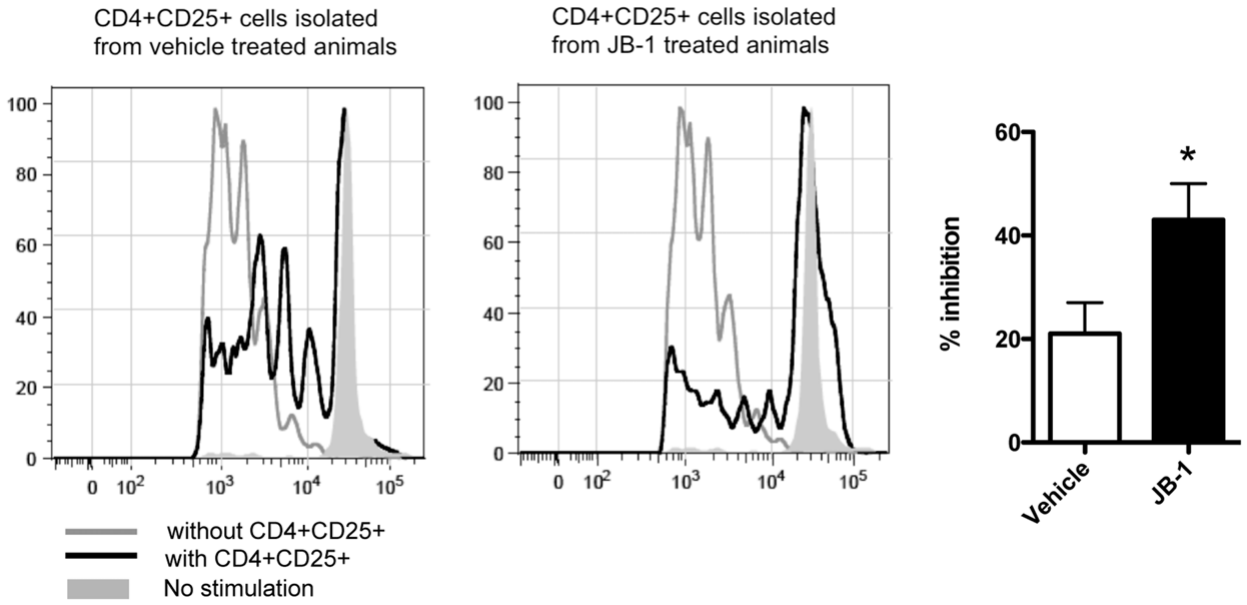
JB-1 enhances the in vitro suppressive capacity of MLN CD4+CD25+ cells. (A) CFSE plots, representative from 4 similar experiments, of the T effector–cell proliferation in the absence (tinted histograms), and presence (open histogram), of T regulatory cells at a Treg to T effector cell ratio of 1∶8 (B) Percentage of inhibition of effector T-cell proliferation by T regulatory cells from treated (JB-1) and vehicle-fed controls. Data presented as mean ± SEM n = 4 *P<0.05. CFSE = carboxyfluorescein diacetate succinimidyl ester.

Having determined that there was an increase in Foxp3 expression in CD4^+^ CD25^+^ cells in the MLN of mice treated with JB-1 we wanted to assess whether this was associated with enhanced suppressor activity indicative of functional T regulatory cells. CD4^+^CD25^+^ cells from the MLN of JB-1 or vehicle-fed mice were freshly isolated ex vivo. These T regulatory cell subsets were co-cultured with CFSE-labeled CD4^+^ CD25^–^ T cells from DO11.10 transgenic mice under activating conditions. Cells from JB-1 treated animals showed a significant (2 fold) increase in their ability to suppress proliferation of activated CD4^+^CD25^–^ cells when compared to vehicle treated controls (Figure 2).

**Figure 3:**
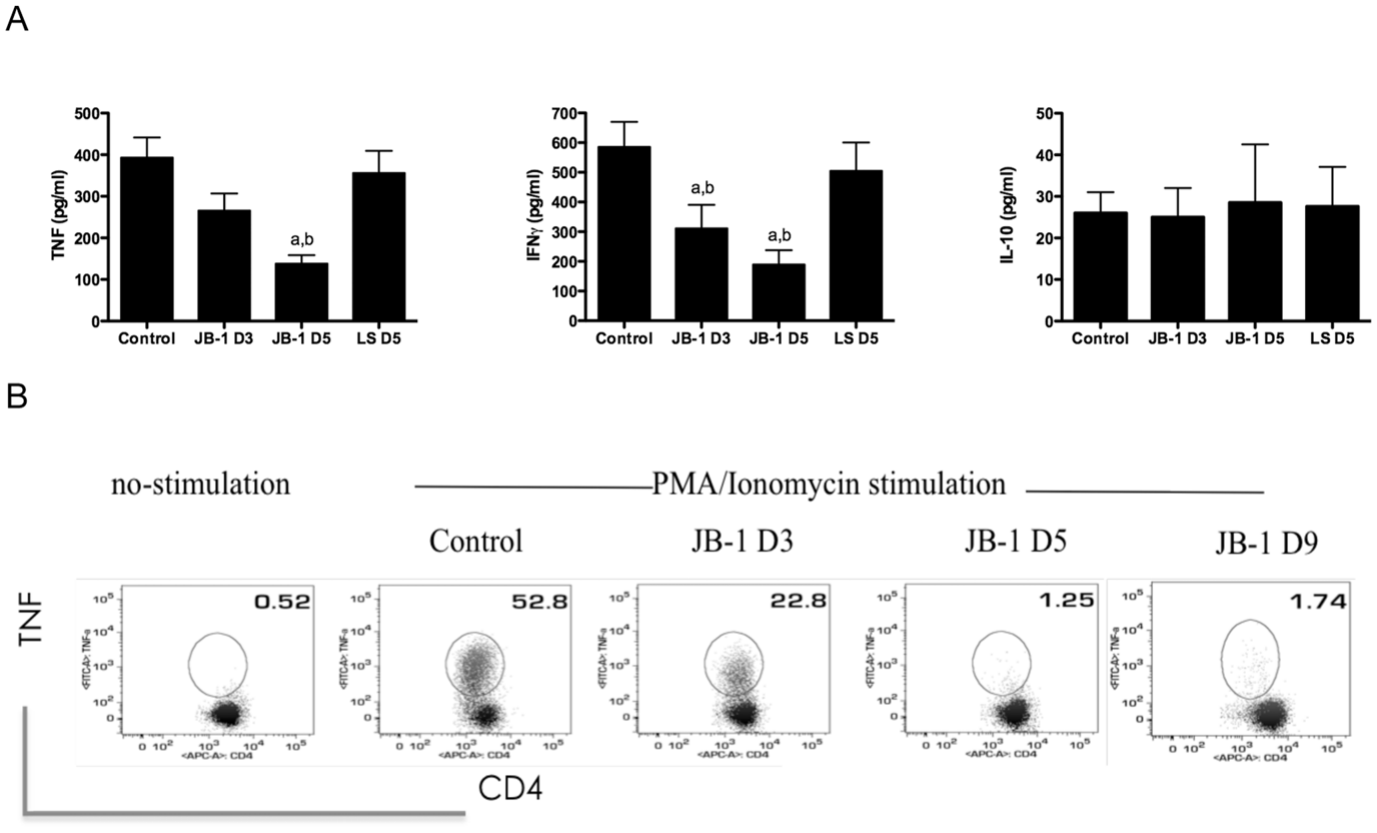
Feeding of JB-1 reduces secretion of inflammatory cytokines by T cells. Balb/c mice were given *L. rhamnosus* (JB-1), *L.salivarius* (LS) or vehicle by gavage for 3 or 5 days. (A) Single cell suspensions of mesenteric lymph nodes were stimulated by antibodies against CD3/CD28 for 48 hr and the supernatants were analyzed for cytokine productions using Cytometric Bead Array Assay. Mean ± SEM values for TNF, IFN-γ and IL-10 release are depicted (n = 6 a, b = P<0.05 compared to vehicle and LS respectively). (B) Single cell suspensions of mesenteric lymph nodes were stimulated by PMA and ionomycin for 4 hr and intracellular TNF by CD4+ CD3+ T cells were depicted data are representative of three similar independent experiments.

The increase in Foxp3+ cells in the MLN was also associated with a corresponding decrease in TNF and IFNγ release following stimulation of isolated MLN cells while IL-10 levels were not significantly altered (Figure 3A). The decrease in TNF and IFNγ was significant following 3 days treatment and maximal at 5 days, corresponding with maximal Foxp3 expression. FACS analysis reveled that TNF production by CD4+ cells was decreased significantly after 3 days treatment and almost abolished at 5 days (Figure 3B). These data suggest the establishment of an anti-inflammatory/tolerogenic environment in the MLN following feeding with JB-1.

### Lactobacillus Exposure Induces Regulatory Dendritic Cells Expressing Heme Oxygenase

**Figure 4:**
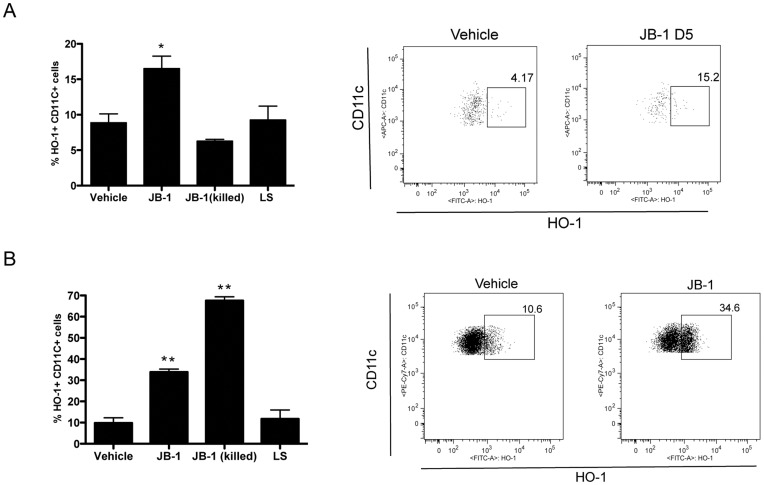
Hemoxygenase-1 expression is up-regulated in dendritic cells following exposure to JB-1. Balb/c mice were given live or killed (gamma-irradiated) *L.Rhamnosus* JB-1, *L.Salivarius* (LS) or vehicle by gavage for 5 days. Single cell suspensions of mesenteric lymph nodes were stained for CD11c, MHCII, and intracellular HO-1 expression using flow cytometry. Shown are representative dot plots and corresponding graphs represents HO-1 (A) expression in mesenteric lymph node DC. (B) Dendritic cells were cultured from bone marrow and CD11c+ cells were purified and cocultured with JB-1 for 24 hr. HO-1 expression in DC was assessed by FACS. Data presented as mean ± SEM n = 5 *P<0.05.

**Figure 5:**
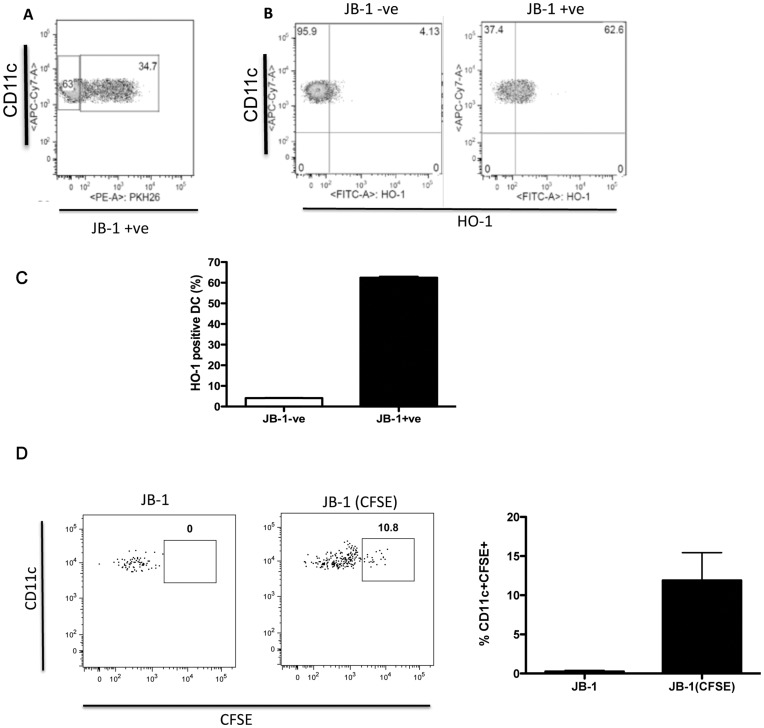
HO-1 expression is enhanced in Dendritic cells assocaited with JB-1. Dendritic cells were cultured from bone marrow and CD11c+ cells isolated and cocultured with PKH26 labeled *L.rhamnosus* JB-1 for 24 hr and HO-1 expression assessed by FACS. A) A dot plot showing a subpopulation of dendritic cells associated with the labeled lactobacillus. This is representative of 4 similar experiments. B) Representative dot plots and C) mean values of the percentage HO-1+ cells in the Lactobacillus associated (JB-1+ve) and non-associated (JB-1-ve) dendritic cell populations. Evidence for in vivo interaction between DC and JB-1 is provided by D) Representative dot plots and E) mean values of CFSE positive cells within the CD11c positive population from Peyers Patches of mice fed with CFSE labeled or unlabeled JB-1. Data presented as mean ± SEM, n = 5, *p<0.05. CFSE = carboxyfluorescein diacetate succinimidyl ester.

Given the evidence that HO-1 can be an important functional component of regulatory DC [23], [26] we investigated the ability of JB-1 to induce HO-1 expression in DC both in vitro and in vivo. Treatment of mice with JB-1 for 5 days lead to a significant increase in CD11c+ cells expressing HO-1 in the MLN (8.9±1.4 vs 17.8±2.2, n = 5 p<0.05) (Figure 4A) and while there was also an increase in HO-1 expression in DC in PP this was not statistically significant (4.6±1.1 vs 7.1±0.6, n = 4 p = 0.1). To determine if JB-1 must be alive to lead to the induction HO-1 we also treated with γ-irradiated bacteria. The killed organisms failed to increase HO-1 in DC within the MLN. The increase in HO-1 could be mimicked by direct co-culture of bone marrow derived DC (BMDC) with JB-1 for 24 hrs (Figure 4B). As with Foxp3 expression in T cells induction of HO-1 was also strain specific as exposure to lactobacillus salivarius, in vivo or in vitro, did not significantly change HO-1 expression in DC (Figure 4). In striking contrast to our in vivo findings, killed JB-1 was even more effective in inducing HO-1 expression in DC, following in vitro exposure, than the live organism (Figure 4B). FACS analysis following in vitro exposure of DC to PKH26 labeled JB-1 indicated that 36.4±1.7% of the DC are associated with JB-1 after 24 h. Within the population of JB-1 associated DC, 61.9±0.6% express HO-1 compared with only 3.9±0.2% of DC not associated with JB-1 (Figure 5) suggesting that direct JB-1/DC interaction is required for the induction of HO-1. To determine whether DC and JB-1 interact in vivo we treated mice with CFSE labeled bacteria. Examination of Peyer’s patches 18 hours following gavage of bacteria revealed a population of CFSE bright DC (11.9±3.5% of CD11c+ cells) which was not observed in vehicle treated mice or mice treated with unlabeled JB-1. This suggests that JB-1 becomes associated with DC in the Peyer’s patches following feeding.

**Figure 6:**
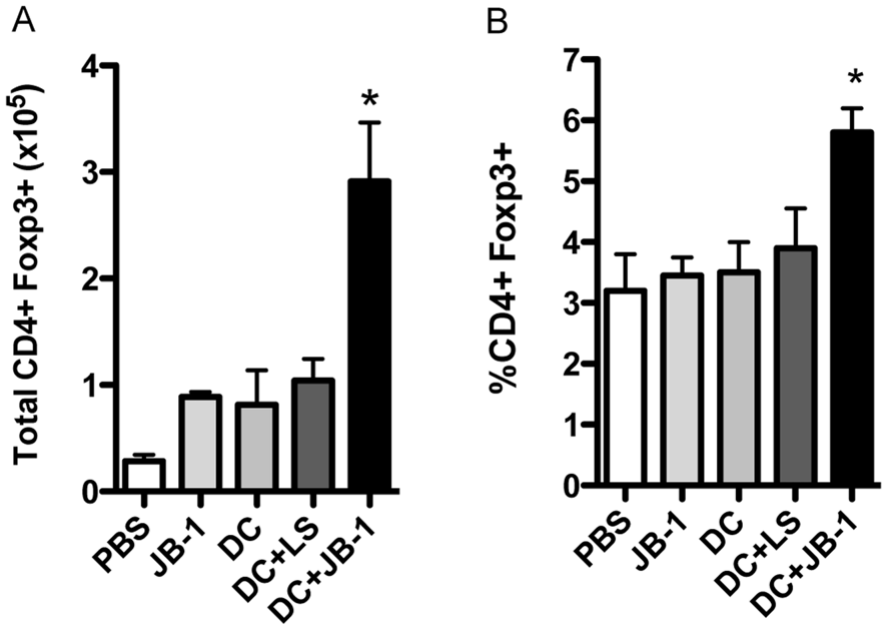
Dendritic cells exposed to JB-1 can induce Foxp3 expression in CD4+ T cells. Numbers (A) and percentage (B) of Foxp3+ CD4+ T cells in the popliteal lymph node of mice 5 days following adoptive transfer of JB-1 loaded BMDC into the footpad (1×10^6^ cells). Transfer of unloaded BMDC served as a control. Data presented as mean ± SEM, n = 5, *p<0.05.

As a test of immunoregulatory capacity, BMDC exposed to JB-1 in vitro were adoptively transferred into the footpad of mice. This lead to a significant increase in CD4+ Foxp3+ cells in the popliteal lymph node, while transfer of the same number of JB-1 alone, unexposed DC or DC exposed to *L. salivarius* had no significant effect on Foxp3 (Figure 6).

### Inhibition of Hemeoxygenase Prevents the Lactobacillus Induced Increase in Foxp3 Expression

**Figure 7:**
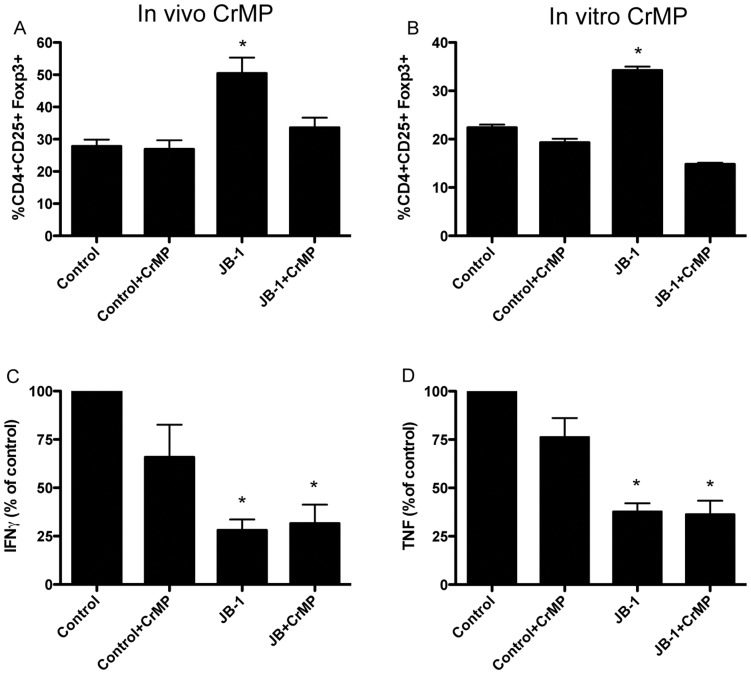
Heme oxygenase-inhibition attenuates Lactobacillus induced foxp3 expression in mesenteric lymph nodes but does not interfere with down-regulation of T cell inflammatory cytokine release. Balb/c mice received i.p. Chromium mesoporpyrin (CrMP) daily for 7 days. Mice were gavaged with JB-1 or vehicle for the last 5 days of experiment and mesenteric lymph nodes were harvested. (A) Single cell suspensions of mesenteric lymph nodes stained for CD4, CD25 and Foxp3 and analyzed by FACS. (B) Isolated mesenteric CD11c+ DC were co-cultured with *L. rhamnosus* JB-1 at a ratio of 10∶1/bacteria:DC, for 2 h at 37°C and incubated in the presence or absence of CrMP (5 µM) with purified CD4+ T cells from DO11.10 transgenic mice for 5 days stained for CD4, CD25 and Foxp3 and analyzed by FACS. (C&D) Single cell suspensions of mesenteric lymph nodes were stimulated by antibodies against CD3/CD28 for 48 hr and the supernatants analyzed for cytokine production. Release of TNF and IFNγ, relative to vehicle control (TNF 398±49.2 pg/ml, IFNγ 592±105 pg/ml) is depicted. Data is presented as mean values ± SEM n = 5, *p<0.05 compared to control values.

There is good evidence that certain immunoregulatory actions of HO-1+ DC are dependent on activity of the enzyme [27], [28]. Therefore we utilized chromium mesoporphyrin (CrMP), a well-characterized HO-1 inhibitor [29], [30] to determine the role of this enzyme in JB-1 mediated changes in the MLN. Treatment of mice with CrMP markedly attenuated the increase in CD4+CD25+Foxp3+ cells induced by JB-1 (Figure 7A). These findings were mimicked in vitro. Isolated MLN DC exposed to JB-1 before co-culture with CD4+ T cells induced an increase in the expression of Foxp3 in the CD4+CD25+ T cell population while addition of CrMP (5 µM) to the co-culture prevented this increase (Figure 7B).

### Inhibition of Heme oxygenase does not Prevent the Lactobacillus Induced Decrease in Inflammatory Cytokine Production

While treatment of mice with CrMP attenuated the increase in CD4+CD25+Foxp3+ cells induced by JB-1, inhibition of HO-1 did not prevent the decrease in IFNγ and TNF production in the MLN (Figure 7C&D).

To further investigate the mechanism underlying this decrease in IFNγ and TNF production we utilized an in vitro system, co-culturing JB-1 exposed DC with CD4^+^CD25^−^ responder T cells from DO11.10 transgenic mice and assessing the effect of blocking signaling of the major regulatory cytokines IL-10 or TGFβ on T cell cytokine production.

**Figure 8:**
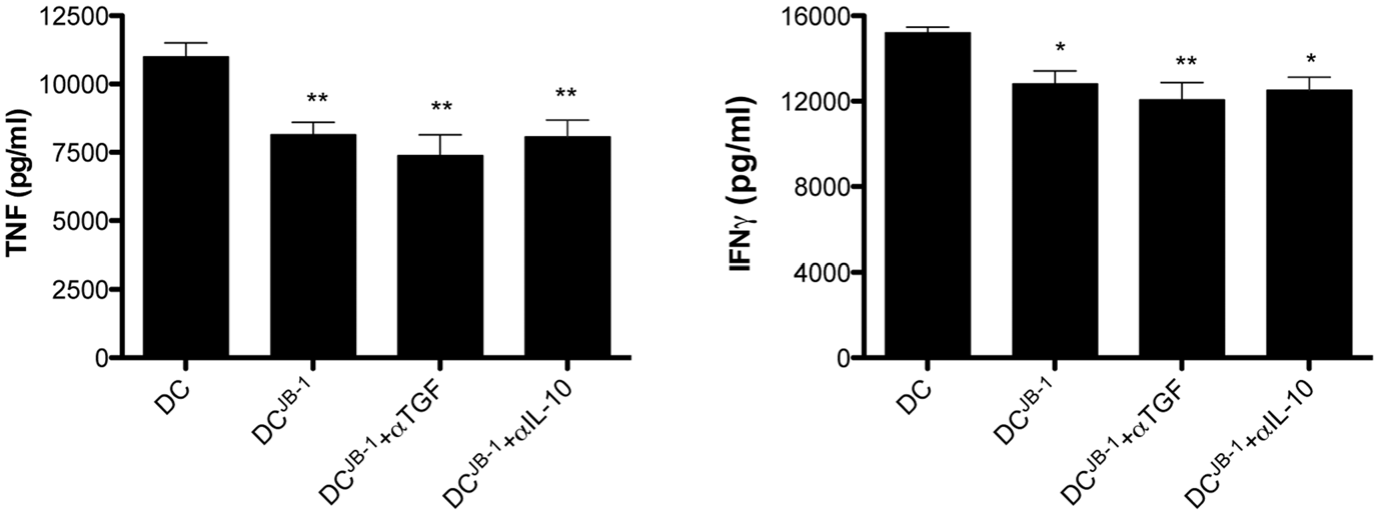
Blocking IL-10 or TGFβ does not prevent the suppression of T cell inflammatory cytokine production by JB-1 exposed DC. Isolated mesenteric CD11c+ DC were co-cultured with *L. rhamnosus* JB-1 at a ratio of 10∶1/bacteria:DC, for 2 h at 37°C and incubated with purified CD4+CD25– responder T cells from DO11.10 transgenic mice for 5 days. T cells were harvested and stimulated by antibodies against CD3/CD28 in the presence of OVA protein for 48 hr and the supernatants analyzed for cytokine production. Where indicated, anti-IL-10 (1 µg/ml) or anti-TGFβ (50 µg/ml) was added to the co-culture. Data is presented as mean values ± SEM n = 5, *p<0.05 compared to control values.

DC isolated from MLN and exposed to JB-1 could mimic effects on T cells observed following feeding decreasing the release of IFNγ and TNF following stimulation (Figure 8). While the decrease in cytokine production was significant the magnitude of the was less than observed in the isolated MLN cells following feeding of the bacteria (Figure 7 C&D) this likely reflects difference between an ex vivo and an entirely in vitro system and the response of transgenic, OVA specific, T cells in contrast to MLN cells. However treatment with neither anti-IL-10 nor anti-TGFβ prevented the JB-1 exposed DC induced reduction of IFN and TNF release by T cells (Figure 8).

Overall these data suggest that HO-1 activity is an important component of Foxp3 induction following JB-1 feeding. However, it is clear that the bacteria trigger additional, HO-1, Foxp3, TGFβ and IL-10 independent, regulatory pathways that contribute to the tolerogenic environment of the MLN.

## Discussion

Recent studies utilizing germ free mice have identified that key species within the microbiota, such as clostridia, single bacterial strains, or components of these strains, can strongly influence the development of T regulatory cells in the intestine [3]–[5]. In the present study we were attempting to gain further understanding of the immunological mechanisms underlying the ability of a single bacterial strain, previously demonstrated to have anti-inflammatory effects, to induce immunosuppression in an adult animal against the background of existing conventional microflora.

It is suggested that the immunomodulatory effect of oral treatment with certain commensal organisms are associated with induction of Foxp3+ Treg and that these cells, induced in the GALT, can spread to sites beyond the intestine in response to immune challenge [12], [13], [18]. This concept is supported by our previous findings that oral treatment with JB-1 results in an increase in Treg in the draining lymph nodes of the lung of OVA sensitized mice following antigen challenge in the airway [12], and by studies demonstrating that Foxp3+ T cell generated in the MLN following exposure to a mixed probiotic population can migrate to sites of inflammation in experimental atopic dermatitis [18]. Here we further investigated the immunomodulatory effects of JB-1. We demonstrated that oral treatment with this bacteria lead to a significant increase in CD4+CD25+Foxp3+ cells in the PP and MLN. The increase in these cells was most marked in the MLN, this is not surprising given its central and critical roles in induction of tolerance/immunity and gating of communication between the gut mucosa and systemic immune system [31]. Interestingly, studies colonizing germ free mice with clostridia [3] or altered Schaedler flora [4] demonstrated regulatory T cell induction in the colonic lamina propria but not in the Peyer’s patches or mesenteric lymph node suggesting immunomodulatory pathways distinct from those observed with JB-1.

The increase in CD4+CD25+Foxp3+ cells is accompanied by a decrease in CD3/CD28 induced TNF and IFNγ production by T cells, with the maximal Foxp3 induction and proinflammatory cytokine inhibition occurring following 5 days treatment. Thus, oral treatment with JB-1 leads to the development of characteristics of a tolerogenic environment in the MLN. Oral tolerance is a key feature of intestinal immunity and generates systemic tolerance to fed antigens. The systemic aspects of tolerance are believed to be mediated by Treg however antigen/DC interaction and the development of regulatory DC phenotype in the MLN is critical to instigating tolerogenesis.

DC are pivotal in early bacterial recognition [17], [32]. Macpherson and Uhr [15] demonstrated that intestinal DC retain small numbers of live commensals for several days, while in vitro, mouse BMDC can take up various lactobacilli by phagocytosis which subsequently influences the ability of the BMDC to produce cytokines [33]. However, few studies have directly linked changes in DC phenotype with Treg induction by commensal bacteria.

Kwon et al [18] reported that administration of a mixture of 5 probiotic strains induced generation of CD4+Foxp3+ Tregs from the CD4+CD25– population. This Treg induction was directly mediated by regulatory DC expressing high levels of IL-10, TGF-β, COX-2, and IDO. Our data supports a similar sequence of events, but demonstrates that a single bacterial strain can induce regulatory DC, primarily in the MLN, that can in turn drive the formation of CD4+CD25+ Foxp3+ cells. In direct contrast to Kwon et al [18] we identified an increase in Foxp3 expression in the CD25+ but not CD25– T cells. One possibility is that there is bacterial strain specific activation of distinct Treg populations. Crucially we identified increased HO-1 expression in DC following treatment with JB-1. Heme oxygenase is an enzyme that catalyzes the rate-limiting step in the degradation of heme to biliverdin carbon monoxide and iron [21]. It has been identified that the inducible isoform, HO-1, is involved in immune regulation [21]. While there have been studies suggesting that HO-1 expression by regulatory T cells plays a critical role in their suppressive activity [34], [35] more recent investigations have disputed this and instead evidence suggests that HO-1 by antigen presenting cells has a more important role in immunosuppression [36], [37]. Overexpression of HO-1 in DC leads to increased secretion of IL-10 and reduced capacity of the cells to trigger effector immune responses [38]. Recently, Mougiakakos et al [39] identified HO-1 expression in human mesenchymal stem cells and demonstrated that pharmacological inhibition of HO-1 activity lead to a significant reduction of the ability of these cells to induce regulatory T cells. George et al. identified that HO-1 expression by antigen presenting cells is required for the function of CD4+CD25+ Treg [22]. DC expressing HO-1 can also directly inhibit T cell proliferation and are involved in tolerance in a rat model of allotransplantation [23]. Similarly it has been reported that hepatic overexpression of HO-1 leads to induction of regulatory T cells that are responsible for increased survival of liver allografts [40]. Here we demonstrate that feeding with JB-1 leads to increased HO-1 expression by DC in the MLN and this effect was mimicked by direct in vitro exposure of bone marrow derived DC to the bacteria. This change in phenotype was associated with increased regulatory activity of these DC as evidenced by the ability to induce Foxp3 expression in lymph nodes following adoptive transfer. Live JB-1 was required to increase HO-1 expression in DC following oral treatment. This finding supports our previous studies showing that killed JB-1 lacked the Treg associated anti-inflammatory action of the live organism in a mouse model of asthma [12], [24]. However, in vitro exposure of killed JB-1 to DC lead to an even greater induction of HO-1 than the live organism. The reasons behind this seeming paradox of in vitro and in vivo responses to the killed bacteria are unclear. However, it is likely that transit through the GI tract is very different for dead and live bacteria and that killed organisms may not reach DC in the Peyer’s patches in significant numbers, if at all. Unfortunately, we were unable to develop an effective method of labelling dead organisms to test this hypothesis within the current study. Similarly, we can currently only speculate on mechanisms underlying the enhanced response of DC to killed versus live bacteria in vitro. It may be that the expression of ligands for pattern recognitions receptors (PRR), such as Toll-like receptors (TLR) and NOD-like receptors (NLR) are altered in the killed bacteria. Alternatively, the γ-irradiation may lead to small changes in the motifs recognized by PRR; it is known that even minor chemical modifications in bacterial cell wall components can result in significantly different immunological consequences [41], [42].

Microbes can also interact with the host immune system by means of their genomic DNA; most notably unmethylated CpG motifs can activate immune responses in vitro and in vivo through activation of TLR9 [43]. DNA is the principal cellular target governing loss of viability after exposure to gamma-irradiation; with cell death predominantly induced by double-strand breaks in DNA that cannot be repaired by the cell [44]. It is possible that such changes in DNA influence immune effects of the bacteria. Clearly further investigations of the differences in the capacity of live and killed JB-1 to induce HO-1 are warranted and may yield important information regarding mechanisms underlying the immunoregulatory actions of certain bacterial strains.

The induction of HO-1 in DC appears to be another example of strain specific immunomodulation by commensal bacteria as the effect of JB-1 on DC was not mimicked by *L.salivarius*. Such strain specific effects are well established. Lyons et al [13] demonstrated differential induction of Foxp3+ T regulatory cells by three bacterial strains was associated with the ability to protect against allergic inflammation in mice. Maassen et al [45] analyzed eight different Lactobacillus strains and demonstrated marked differences with respect to induction of pro-and anti-inflammatory cytokines in the gut mucosa. Similarly, Christensen et al. [46] showed that various lactobacillus species differentially stimulated mouse BMDC, with some strains strong inducers of IL-12 and TNF while others were weak inducers. It is still unclear exactly what underlies the distinct immunomodulatory effects of seemingly similar bacteria but small changes in only one microbe associated molecular pattern can result in significant changes in the immune response to bacteria. A study by Grangette et al [42] demonstrated that a mutant strain of L. plantarum (Dlt-) that incorporates less d-Alanine in cell wall teichoic acids had a markedly reduced ability to induce secretion of proinflammatory cytokines from peripheral blood mononuclear cells while stimulating increased IL-10 production.

The observed increase in HO-1 expression occurred almost exclusively in those DC associated with JB-1 or components of the bacteria, suggesting that direct interaction of the bacteria and DC is required to mediate the phenotypic and functional changes and indeed we observed uptake of CFSE labelled JB-1 by DC in the Peyer’s patches demonstrating that such interaction occurs in vivo. The fact that we did not detect statistically significant increases in HO-1 expression by DC within the Peyer’s patches may suggest that, upon phagocytosis of JB-1, DC migrate quickly to the MLN and that while induction of HO-1 is triggered within the Peyer’s patches it develops over time as the DC migrate.

The DC receptors that might be involved in mediating these effects are currently unknown. Smits et al [8] demonstrated that certain lactobacilli that induced T cells to produce IL-10 when cultured with human monocyte-derived DC bound the C-type lectin DC-specific intercellular adhesion molecule 3-grabbing non-integrin (DC-SIGN). Blocking antibodies to DC-SIGN inhibited the induction of the Treg cells by these lactobacilli, indicating that ligation of DC-SIGN can actively prime DC to induce Treg cells. However there is good evidence that additional receptors including toll-like receptors 2,4 and 9 are critical to the tolerogenic and/or anti-inflammatory effects of probiotics [24], [33], [43], [47], [48] and it is likely that multiple receptors are involved in determining differential immunological responses following exposure to specific bacterial strains.

It has been demonstrated that inhibitors of TGFβ, COX-2 or IDO can prevent DC induced Foxp3 expression in the CD4+CD25– cell population following treatment with a mixed strain probiotic preparation, suggesting that multiple components of regulatory DC are required to influence Treg generation [18]. Through use of the heme oxygenase inhibitor, chromium mesoporphyrin, we have now identified heme oxygenase as playing an important role in induction of CD4+CD25+Foxp3+ Treg. Whether differential expression of regulatory mediators in DC determines bacterial strain specific induction of Treg phenotypes remains to be determined.

Interestingly, while inhibition of heme oxygenase prevented the increase in Foxp3 expression by CD4+CD25+ cells, in vivo and in vitro, it did not alter the inhibition of TNF and IFNγ production by T cells induced by JB-1. In addition the presence of either anti-IL10 or anti-TGFβ did not modulate this inhibition of inflammatory cytokine production. These results suggest that the increase in Foxp3 expressing Treg is independent of the associated decrease in inflammatory cytokine production and that while HO-1 represents a component of the immunoregulatory pathway leading to the induction of Foxp3+ expression it does not influence effector T cell activity in the MLN. Thus it appears that JB-1 feeding triggers multiple regulatory pathways. The nature of these additional regulatory pathways remains to be determined but could include direct suppressive action of DC through COX-2 or IDO mediated responses [18] or the induction of a Foxp3- population of regulatory T cells.

Our findings indicating role for HO-1 in Foxp3 induction but not in suppression of inflammatory cytokine production by CD4+ T cells contrasts those of Moreau et al [23] which suggested that, in vitro, HO-1 activity of DC does not play a role in the stimulation and expansion of Treg in vitro but instead directly inhibits T cell proliferation. However there are a number of in vivo studies that clearly support a role for HO-1 in promoting Foxp3+ Treg, [28], [47], [48] for example Xia et al showed that HO-1 activity is required for up-regulation of Foxp3+ Treg and subsequent attenuation of ovalbumin induced airway inflammation induced by the heme oxygenase activator, hemin [49].

It may be that in vivo, expression of HO-1 by other cells types is involved in promoting Foxp3 expression but it is clear from our adoptive transfer experiments that JB-1 exposed DC do have the ability to induce Foxp3 expression.

In conclusion, we have demonstrated that oral treatment with JB-1 induces a tolerogenic environment in the MLN that includes the generation of HO-1+ regulatory DC. Furthermore, we have identified heme oxygenase activity as an important mediator of Foxp3+ Treg induction by this bacteria. However, additional, as yet unknown, regulatory pathways are involved in JB-1 mediated down-regulation of inflammatory cytokine production in the MLN. Given the extant literature suggesting a protective effect of increased HO-1 expression in models of asthma and inflammatory bowel disease [28], [48], [50]–[55], disorders that are also suggested targets of microbial based or probiotic therapies, it will be interesting to determine whether activation of this enzyme is an important element of beneficial effects described for certain commensal organisms in these systems.

## Materials and Methods

### Animals

These experiments were performed in strict accordance with guidelines of the Canadian Council for Animal Care. Protocols were approved by the Animal Research Ethics Board of McMaster University (approval number 09–05–17). Adult male Balb/c and DO.11.10 transgenic mice (20–25 g) were maintained in an automatic light/dark cycle (light periods of 12 h) and provided water and chow ad libitum. Mice were acclimatized to the animal facility for 1 week before experimentation. Age-matched (8–9 wk old) animals were used in all experiments.

### Bacterial Treatment

Two bacterial strains were used in this study, *L. rhamnosus* (JB-1), is the same as that employed in several published investigations [12], [24], [25] and was previously referred to as *L reuteri*. This strain was recently confirmed as a *Lactobacillus rhamnosus*, by AFLP fingerprinting and full genomic analysis. It was identified as a strain distinct from L rhamnosus GG [56], and any of the 118 L rhamnosus strains, examined by Vancanneyt et al. [57] and does not belong to any of the 7 clusters identified by the Bacteria Collection Laboratory for Microbiology, University of Ghent, Belgium. *L. salivarius* UCC118 were a gift from Dr. B. Kiely (Alimentary Health, Cork, Ireland). Both strains were prepared from frozen stocks (–80°C) as described previously [24]. Briefly, bacteria were suspended in Man-Rogosa-Sharpe liquid medium (MRS broth; Difco Laboratories, Detroit, MI) plated in MRS agar, cultured anaerobically at 37°C for 24 hours, then inoculated in fresh MRS broth and grown at 37°C under anaerobic conditions for 48 hours. Bacteria were washed twice with sterile phosphate-buffered saline (PBS) to a concentration of 6×10^8^ bacteria/ml as determined by a Vitek colorimeter (bioMérieux, Hazelwood, MO). Bacterial suspensions were then centrifuged and resuspended in MRS broth to give a concentration of 5×10^9^/ml^24^. Suspensions of L. rhamnosus JB-1 were killed by γ-irradiation with Cobalt 60 for 20 hours at 8.05 Gy/minute. The resulting viability was determined by plating on MRS agar plates under anaerobic conditions for 72 hours at 37°C. No bacterial growth was detected in irradiated preparations after 72 hours of culture.

Naïve mice received 1×10^9^ JB-1 or *L.salivarius* in 200 µl of MRS liquid medium broth via oral gavage needle daily for 3, 5 or 9 days. Control animals were treated daily with 200 µl of MRS broth alone. Following treatments animals were euthanized and Peyer’s Patches and MLN removed and processed for FACS analysis or cell isolation.

### Inhibition of Heme oxygenase

Heme oxygenase activity was inhibited in vivo using the potent and specific heme oxygenase inhibitor [30] Chromium(III) Mesoporphyrin IX chloride(CrMP) (Frontier Scientific Inc.). 25 mg/kg of CrMP was administered (i.p) daily over a period of 7 days starting 2 days before mice were gavaged with bacteria or MRS broth.

### Labeling of Bacteria

Red fluorochrome PKH26 (Sigma-Aldrich, Okville, Canda) was used to label bacteria according to the manufacturer’s instructions. Briefly, 2×10^8^ bacteria were washed with PBS and the cell pellet was dissolved in PKH-26 diluent. PKH-26 fluorochrome was diluted in a separate tube in PKH-26 diluent. The bacterial suspension was added to the PKH-26-containing tube and incubated for 7 min at room temp. Staining was stopped by adding PBS containing fetal calf serum for 2 min. Bacteria were then washed three times with PBS and resuspended to the desired concentration in serum- and antibiotic-free medium prior to incubation with DC.

For in vivo studies bacteria were labeled with carboxy fluorescein diacetate succinimidyl ester (CFSE) as described previously [58], with some modifications. Briefly, 5×10^9^ bacteria were washed with PBS and CFSE was added to the tube at final concentration of 5 µM and incubated for 10 min under constant shaking at room temperature. Staining was stopped by adding PBS containing FCS for 2 min. Bacteria were then washed three times with PBS, 5% FCS and resuspended in PBS at 5×10^9^/ml prior to gavage.

### Culturing BMDC

Bone marrow cells were cultured in the presence of granulocyte monocyte colony stimulating factor for 7 days as previously described [59], [60]. The cells were fed with fresh medium and cytokine on days 3 and 6 of the culture. On day 7 of culture DC were used for performing the experiments. Cell viability was assessed using trypan blue stain prior to and following co-culture with bacteria.

### Cell Isolation

CD4+CD25+ cells were isolated from mesenteric lymph nodes of bacteria or vehicle fed BALB/c mice to 89% purity using MACS bead mouse Treg isolation kit (Miltenyi Biotec,Auburn,CA). CD4+CD25– T cells were isolated from DO.11–10 transgenic mice to a purity of 94%. DC were isolated from mesenteric lymph nodes of naïve wild type mice using Miltenyi Biotec CD11c Microbeads.

### Adoptive Transfer of Dendritic Cells

Bacteria were co-cultured with BMDC at a ratio of 10∶1/bacteria:DC, for 24 hr at 37°C. DC were washed three times with PBS and viability assessed with trypan blue stain prior to injection into the footpad (1×10^6^ cells). This dose was chosen on the basis of previous studies [59]. Unloaded DC and bacteria alone served as controls.

### T-regulatory Cell Suppressive Function

To examine the suppressive capacity of JB-1 induced T-regulatory cells, we used the in vitro assay of T-regulatory cell activity as described previously [12]. Briefly, T regulatory cells (Miltenyi Biotec) were isolated from mesenteric lymph nodes of JB-1- or vehicle-MRS broth fed mice. This cell subset (6.25×10^3^ ) were cultured with CD4+CD25– responder T cells isolated from DO11.10 transgenic mice (5 × 10^4^ ) for 4 days in U-bottomed 96-well plates at a ratio of 1 Treg to 8 CD4+CD25– responder T cells in the presence of soluble anti-CD3 (1 µg/ml) and BM-DC (5 × 10^3^ ). Prior to the co-culture assay, DC were pulsed with 1 mg/ml OVA protein for 16 h followed by a 20 min incubation with 50 µg/ml mitomycin C in a 37°C, 5% CO_2_ humidified incubator. Responder T cells were labeled with carboxy fluorescein diacetate succinimidyl ester (CFSE). Proliferation of CD4+ T cells from DO11.10 mice, detected by KJ1–26 mAb specific for the transgenic TCR expressed by DO11.10 T cells, was assayed by CFSE dilution using FACS analysis and proliferation calculated using FloJo version 9.5.2 software (TreeStar, Ashland, OR).

### T cell Proliferation and Stimulation

Isolated mesenteric CD11c+ DC (Miltenyi Biotec ) were co-cultured with bacteria at a ratio of 10∶1/bacteria:DC, for 2 h at 37°C and after extensive washing were incubated with purified CD4+CD25– responder T cells from DO11.10 transgenic mice for 5 days in U-bottomed 96-well plates in the presence of soluble anti-CD3 (1 µg/ml) and 1mg/ml OVA protein. Where indicated, anti-IL-10 (1 µg/ml, BD Pharmingen), anti-TGFβ (50 µg/ml, R&D Systems) or CrMP 5 µM was added to the culture. On day 5 of culture T cells were harvested and replated into anti-CD3 (10 mg/ml) pre-coated 96-well cell culture plates and at a density of 1×10^6^ cells/ml with soluble anti-CD28 (2 mg/ml) for 48 hours. Supernatants were collected and stored at −80°C before cytokine quantification.

### FACS Analysis

Cultured DC or single cell suspensions from PP or MLN were stained as described previously [12], [61]. with different extracellular markers including CD11c, CD3, CD4, CD25 (BD Pharmingen, San Diego, CA) and intracellular IL-10, Foxp3 (eBiosciences, San Diego, CA), and HO-1 (Abcam, Cambridge, MA). For intracellular staining, the cells were initially stained for surface markers and then fixed, permeabilized and stained for intracellular expression of the markers as recommended by the related manufacturer. For intracellular cytokine expression, cells (1×10^6^ cells/ml) were stimulated by plate bonded anti-CD3 and soluble anti-CD28 in 96-well cell culture plates and were incubated for 6 h in the presence of the protein transport inhibitor GolgiStop™ (BD Biosciences) prior to the staining [61]. Data were acquired with LSRII or FACSCanto (Becton Dickinson, Oakville, Canada) and analyzed with the FlowJo program (TreeStar, Ashland, OR). Absolute numbers of cells were calculated by multiplying the percentage of positive staining cells in the total acquired events by the total number of cells isolated from the tissue analyzed.

### Cytometric Bead Array Assay

96-well cell culture plates were coated with anti-CD3 (10 µg/ml) overnight. Cells were then plated at a density of 1×10^6^ cells/ml with soluble anti-CD28 (2 µg/ml) for 48 h. Supernatants were collected and stored at −20°C prior to cytokine quantification. Cytokines in supernatants were analyzed with the mouse flex set cytokine cytometric bead array kit (Becton Dickinson, Canada) and BD FACSArray bioanalyzer.

### Statistical Analysis

Experimental results are expressed as means ± the standard errors of the means. Statistical analyses were performed by means of one-way analysis of variance (ANOVA), followed by Tukeys test for comparing all pairs of groups. Significant differences between two groups were determined using the unpaired Student's t test. A statistical software package (GraphPad PRISM™ version 5.0) was used for the analysis. A p value of less than 0.05 was considered statistically significant.
